# Successful management of a complicated clinical crisis: A patient with left-sided endocarditis and secondary hemophagocytic lymphohistiocytosis: a rare case report and literature review

**DOI:** 10.1097/MD.0000000000009451

**Published:** 2017-12-22

**Authors:** Peipei Xu, Hui Zeng, Min Zhou, Jian Ouyang, Bing Chen, Qiguo Zhang

**Affiliations:** Department of Hematology, The Affiliated Drum Tower Hospital, Nanjing University Medical School, Nanjing, People's Republic of China.

**Keywords:** daptomycin, hemophagocytic lymphohistiocytosis, infectious endocarditis, methicillin-resistant *Staphylococcus epidermidis*, multidisciplinary team

## Abstract

**Rationale:**

Hemophagocytic lymphohistiocytosis (HLH) secondary to methicillin-resistant *Staphylococcus epidermidis* (MRSE)-related left-sided infectious endocarditis had never been reported before. In the last decade, daptomycin, a novel lipopeptide antibiotic, showed its excellent role in anti-Gram-positive bacteria, including soft tissue infection, bloodstream and deep tissueinfection.

**Patient concerns:**

An Asian women under sever condition due to the cooccurrence of HLH and MRSE-related endocarditis while also be allergic to vancomycin. The patient was cured by high-dose daptomycin monotheraphy, HLH-2004 protocol and cardiothoracic surgery to remove the valve at last, and was obviously benefit from the endeavor of a multidisciplinary team (MDT) strategy.

**Diagnoses:**

IE was made on March 27according to the modified Duke criteria. HLH was diagnosed too.

**Interventions:**

The patient was cured by high-dose daptomycin monotheraphy, HLH-2004 protocol and cardiothoracic surgery to remove the valve at last, and was obviously benefit from the endeavor of a multidisciplinary team (MDT) strategy.

**Outcomes:**

The patient was healthy andstable when we published this case.

**Lessons:**

This case proves high-dose daptomycin monotheraphy could be used as an effective alternative regimen for vancomycin in treating MRSE-related left-sided endocarditis and highlight the importance of early diagnosis and appropriate management for HLH. Furthermore, our work suggests an MDT model as a practical strategy in managing similar clinical situation.

## 1 Introduction

Infectious endocarditis (IE) is a potentially fatal disease, and the treatment involves the administration of antibiotics and surgery to remove the vegetation, if necessary. Hemophagocytic lymphohistiocytosis (HLH), also known as hemophagocytic syndrome (HPS), is a rare condition with an immediate death risk, which leads to a multisystem inflammation and organ infiltration. Partial HLH cases can be cured by chemotherapy, if they are treated in time. However, chemotherapy also suppresses the immune system, and when HLH is associated with a severe uncontrolled infection, early chemotherapy could be a contradiction as it may deteriorate patient's condition and cause consequential problems.

Daptomycin is an antibactericidal agent, which is effective against a wide range of Gram-positive bacteria. It was first approved by the US Food and Drug Administration in 2006 for the treatment of bloodstream infections, including right-sided infective endocarditis (IE) (6 mg/kg/d), as well as for complicated skin and soft tissue infections (4 mg/kg/d).^[1–3]^ The antibacteria efficacy dependents on its pharmacodynamic concentration and penetration degree of the bacterial biofilm, and it may help to prevent the emergence of bacterial resistance. Hence, high-dose (>6 mg/kg/d) daptomycin is considered an alternative option in treating difficult-to-treat infections or deep-sited Gram-positive infections. In this article, we report, for the first time, a clinical case of methicillin-resistant *Staphylococcus epidermidis*-related IE complicated with secondary HLH, in which the patient was cured with high-dose daptomycin monotherapy, according to the latest guidelines,^[4,5]^ steroid and chemotherapy, according to HLH-2004 protocol,^[6]^ and collaborative management from multiple departments.

## 2 Case presentation

The patient provided informed consent for data analyses in compliance with the Declaration of Helsinki and the Ethics Committee of our hospital.

### 2.1 Brief history

A 40-year-old Asian woman was admitted to the infectious disease unit of our hospital with uncontrolled repeated fever for >1 month. Prior to this admission, the patient was diagnosed as subacute liver failure of unknown cause on February 15, 2015. The local hospital treated her with artificial liver support and intravenous hepatoprotectants. The patient responded well, lab results showed transaminases and serum bilirubin improved.

During liver-protecting treatments, the patient developed a fever, with no signs of localized infection. Patient’ body temperature peaks fluctuating between 39°C and 40°C. The first set of blood cultures was negative, and a Computed tomography scan demonstrated bilateral pleural effusion. The complete blood count (CBC) revealed hemoglobin (HB) 65 g/L. A bone marrow biopsy demonstrated suppressed erythroid hematopoietic function and increased granulocytes with immature morphology. Considering the patient's persistent fever and abrupt drop in HB, hemophagocytosis lymphohistiocytosis was suspected, and soluble cell surface antigen (CD)25 was tested.

### 2.2 Physical examination

The patient appeared to be pallor and frail, T 36.5°C, BP108/68 mm Hg, P 98/min, and R20/min. Mild jaundice in skin and sclera. A grade 4 systolic murmur was detected in the cardiac auscultation of the aortic focus. Palpation showed splenomegaly with the lower edge 4 cm below the left costal margin.

### 2.3 Lab results and main diagnosis

The initial blood samples showed anemia (a hemoglobin value of 53 g/L) and a white blood cell count of 5.0 × 10^9^ /L, with a differential of 76.8% neutrophils and platelet (PLT) 147 × 10^9^/L. The biochemical tests showed normal kidney function, slightly elevated transaminases and significantly increased total bilirubin. The serum ferritin level was 674 mg/L. Coagulation profile showed the fibrinogen, 1.5 g/L. Lymphocyte immunophenotype (subsets) showed an NK-T cell percentage of 1.4%. Common virus screening, including human hepatitis virus, cytomegalovirus, virus, and human immunodeficiency virus, were negative. The urinalysis showed 1+ urinary bilirubin. A repeated bone marrow biopsy performed on the 1st hospital day showed an increased ratio of polymorphonuclearleukocytes but no signs of hemophagocytosis.

**Figure 1:**
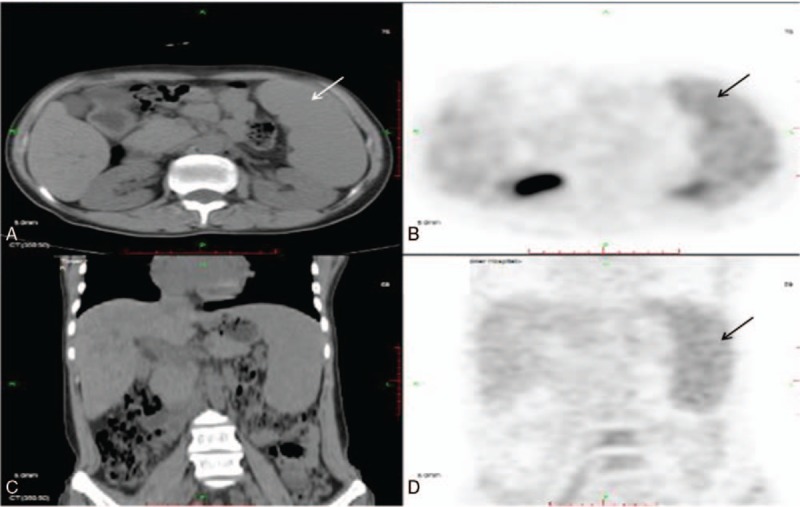
PET-CT on March 27, 2015. (A, B, and D) Splenomegaly and a relatively increased SUV (maximum at 3.3) of the spleen,compared with the liver, was observed. (C and D) No mass or sign of metastasis was observed in the abdominal cavity. The 3 arrows in (A, B, and D) all point at the enlarged spleen.

The 3rd hospital day, the result of soluble CD25 was 15386 U/mL (334–710 U/mL). A full body positron emission tomography-CT scan demonstrated hepatosplenomegaly and relatively increased standard uptake value of the spleen, with no signs of malignancies (Fig. 1). The same day 2 sets of blood cultures, including 1 before admission, were both positive for methicillin-resistant *Staphylococcus epidermidis* (MRSE).

**Figure 2:**
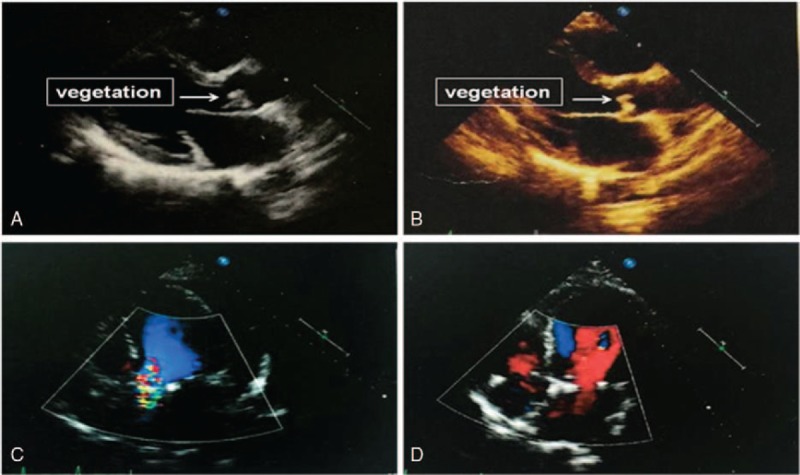
Echocardiography images during hospitalization. (A and B) Echocardiography images obtained on April 10, 2015 and May 7, 2015, where arrow point at revealed a well-shaped vegetation attached to the aortic valve. (C and D) The images obtained on June 4, 2015, 1 week after surgery, and September 08, showed no left-to-right atrial shunt after surgery.

Meanwhile, a diagnosis of IE was made on March 27 according to the modified Duke criteria.^[7]^ Two weeks later, cardiac ultrasound images showed a completely well-shaped vegetation attached to the aorta (Fig. 2),which confirmed the diagnosis of definite IE.

Even though the diagnosis of endocartidis was clear, the patient still progressed over time. Routine blood tests revealed a deteriorating condition and pancytopenia. On April 2, a CBC showed white blood cell (WBC) 3.2 × 10^9^/L, HB 65 g/L, and PLT 92 × 10^9^/L. After consultation with the hematology department, HLH was diagnosed. Table 1 shows our diagnostic basis. Considering there was no relevant family history but a documented infection, the patient was categorized as having an infection-associated secondary form of HLH infection-associated HPS.

**Table 1:**
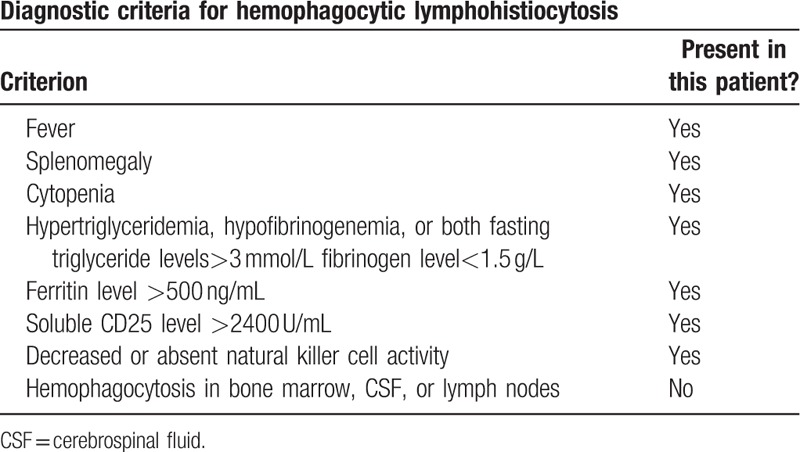
At least 5 of the 8 criteria must be present for a diagnosis to be established.

### 2.4 Treatment course

According to the patient's blood cultures results, intravenous vancomycin 0.5 g was administered every 8 hours. However, rash appeared indicating an allergic reaction. Thus, vancomycin was substituted with teicoplanin the next day. Meanwhile, treatment with intravenous dexamethasone (10 mg/m^2^) and large-dose intravenous immunoglobulin (IVIG) (20 g/d) was started after the diagnosis of HLH was made. However, fever was not under control and 3 separate sets of blood cultures obtained on April 6 to 8 were all confirmed as MRSE (+) (Fig. 2). Bone marrow culture obtained on April 08 was also positive for MRSE.

Meantime the CBC revealed a significantly increased eosinophil count, which may indicate anaphylaxis. On April 9, high-dose Intravenous daptomycin was initiated at a dose of 10 mg/kg every 24 hours (500 mg/d) and was continued for 9 weeks. As a result, the fever was gone and no adverse event was found.

Although after the dexamethasone and IVIG, repeated CBC showed hemoglobin level kept decreasing. On April 12, some of the values even hit the nadir, as follows: BP: 90/49 mm Hg, WBC 1.4 × 10^9^/L, PLT 30 × 10^9^/L, HB 57 g/L, and Fig 1.2 g/L. A multidisciplinary team (MDT) meeting was called to evaluate this critical situation. Hematologist suggested adding Ciclosporin A and etoposide16 to the current treatment. The cardiothoracic surgeon confirmed the surgical indication and suggested an immediate operation when the patient is stable. With targeted antibiotic treatment and chemotherapy, the patient's general condition gradually improved, the patient's laboratory tests were consistent with the recovery.

**Figure 3:**
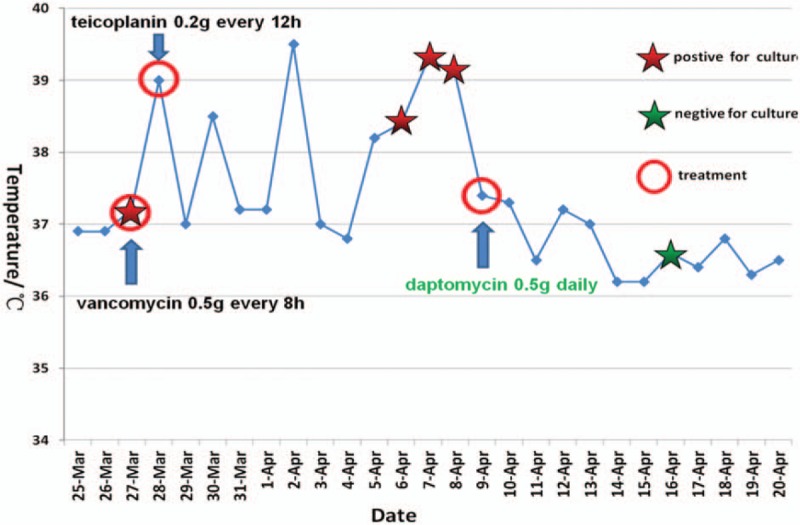
Shows how the patient's temperature responded to antibiotics during the treatment course and the different results of the culture with time. Note: the red stars indicate that bacterial colonies of methicillin-resistant *Staphylococcus epidermidis* were observed in the blood or bone marrow samples, and the green star indicates that no living microbes were detected in the blood sample.

One week after daptomycin was initiated, a repeat blood culture obtained was found to be sterile. The complete blood count, coagulation function, and liver function was also normalized (Fig. 3). The patient was transferred to the cardiothoracic surgery department. Surgery was performed, and the vegetation on the aortic valve was successfully removed. The subsequent culture of the vegetation specimen was later confirmed as negative, the patient steadily improved postoperatively and was discharged on June 12, 2015.

During following up the echocardiography was repeated and the results are shown in Figure 2. The patient was healthy and stable when we published this case.

## 3 Discussion

This case report is the first description of a complicated condition in which the patient concurrently developed MRSE-related left-sited endocarditis and acquired HLH. The patient was cured by high-dose daptomycin monotherapy and chemotherapy according to HLH-2004 protocol, and was obviously benefitted from multidisciplinary teamwork.

### 3.1 Daptomycin

Daptomycin is active against most Gram-positive organisms, such as *Staphylococci*, *Streptococci*, and *Enterococci*. The mechanism occurs via its binding to the bacterial membranes of cells, both during growth and in the stationary phase, which causes depolarization and leads to the rapid inhibition of protein and nucleic acid synthesis, hence causing bacterial cell death.^[8]^ It has already been licensed to treated *Staphylococcus aureus* bacteremia and right-sided endocarditis, and complicated skin and skin-structure infections caused by Gram-positive pathogens.^[2,3,9]^ In 2013, an observational multicenter prospective cohort study conducted by Carugati et al^[10]^ showed that daptomycin resulted in significantly faster bacteremia clearance, compared to the standard-of-care among patients with MRSA, which maybe 1 of the reasons why there is little resistance to daptomycin. Recently, Guleri et al^[11]^ published a study (European Cubicin Outcomes Registry and Experience) showing that daptomycin is effective for the treatment of left-sided IE (LIE) and both right- and left-sided IE, in addition to RIE caused by Gram-positive bacteria, including MRSA. The latest guidelines for the management of IE from Europe^[5]^ and North America^[4]^ both recommend that high-dose daptomycin (≥10 mg/kg once daily and ≥8 mg/kg/dose separately) can be used as an alternative therapy to vancomycin for treating native valve endocarditis caused by methicillin-resistant *Staphylococci*. Additionally, the former study noted that daptomycin should be given at high doses and combined with beta-lactams to increase its activity and avoid the development of resistance.^[2,12]^ In this case, we used high-dose daptomycin monotherapy to treat an Asian woman with MRSE-related left-sided native valve endocarditis. Additionally, even though the duration of treatment with daptomycin was longer than previously reported, there was no resistance been found. We did not use daptomycin combined with beta-lactams due to the financial burden of the patient, but it turned out high-dose daptomycin monotherapy was successful, and there were no adverse events noticed during the treatment course.

### 3.2 MRSE

IE is a relatively rare infectious disease with an annual incidence ranging from 3 to 7/100,000 person-years, according to the latest population surveys.^[13–15]^ Nearly 41% of natrual valve endocarditis (NVE) cases are caused by methicillin-resistant strains, and these resistant strains are recognized to be closely related to health care-associated infections.^[16]^ However, less than 10% of all NVE cases, excluding those related to intravenous drug use, are caused by the most common coagulase-negative Staphylococci (CoNS), mostly *Staphylococcus epidermidis*.^[16]^ Though CoNS, such as MRSE, are a common cause of nosocomial bloodstream infections and an emerging cause of NVE, there is little published information regarding the efficacy of daptomycin against these pathogens. This case is considered as health care-associated because the patient, who denied any history of drug abuse, developed a fever in a medical institution after an invasive operation during artificial liver support treatment. The optimistic outcome of this case is consistent with that obtained in a recent experimental study,^[17]^ in which the researchers used rabbit model with human-adapted pharmacokinetic and proved daptomycin at doses of 6 or 10 mg/kg/d are more effective than vancomycin for the treatment of experimental endocarditis due to MRSE.As such, daptomycin may be offered as an alternative in the limited database of clinical treatments available for MRSE-related LIE.

### 3.3 HLH

The routine treatments for HLH are glucocorticoids, IVIG, chemoimmunotherapy and allogeneic hematopoietic stem cell transplantation, based on the latest guidelines of Histiocyte Society (6). For infection-associated HLH, treatment goals include suppress the severe symptoms caused by hyperinflammation and kill pathogen-infected antigen-presenting cells, remove the stimulus for the proceeding but ineffective T-cell activation. Pathogen-directed antimicrobial therapy is usually insufficient to suppress hyperinflammation, hence, we need glucocorticoids and chemoimmunotherapy in certain cases, but the immunosuppressive therapy should be applied with caution because the infection could deteriorate if the patient's immune system is impaired. There have been 4 clinical cases^[18–21]^ reported about endocarditis-associated HPS, pathogens are *Mycobacterium tuberculosis*, Abiotrophia defective, Group G Streptococci (with coinfection by cytomegalovirus) and *Histoplasma Capsulatum* (with coexistence of MRSA bacterimia), respectively, 1 in a pediatric patient, the other 3 in adults. Among these 4 patients, 1 adult did not survive before formal treatment whose diagnosis was based on autopsy, others are cured by pathogen-targeted therapy for IE. None of the 4 cases deliver a storm like battle between life-threatening infection and immunosuppressive status induced by a full protocol for HLH. The case presented here is a rare condition of HLH secondary to MRSE-related IE, which has never been reported. In this case, an initial therapy with IVIG and dexamethasone for 8 weeks was started right after diagnosis, but the disease progressed over time. Thus, etoposide and cyclosporine A were added, and the antimicrobial agent was changed to a more effective 1. Finally, the patient recovered from severe cytopenia and poor coagulation function and remained stable preoperatively. This case offered us the experience of treating infection-associated HLH with an uncontrolled underlying infection. Specifically, if a patient has a severe infection and also HLH is suspected, standardized and individualized treatment for HLH should be given as soon as possible. Additionally, physicians should emphasize the importance of timely and efficient treatment of the primary infection.

In conclusion, we report the first case of the successful management of a patient with MRSE-related IE complicated with secondary HLH. We have shown that high-dose daptomycin monotherapy is an effective and safe method for treating MRSE-related IE patient who result in secondary HLH when vancomycin and teicoplanin are not viable options. Most importantly, our work has shown the necessity of MDT model in treating severe, complicated diseases for which there is little former experience.

## Acknowledgments

We would like to thank the volunteer who took part in this study. This work was supported by the National Natural Science Foundation of China (81400162, 81570174), the Jiangsu Provincial Medical Youth Talent (QNRC2016039), and the Technique Development Foundation of Nan Jing (Outstanding Youth Foundation, JQX1500 and YKK16099).

## References

[1] Arbeit RD Maki D Tally FP The safety and efficacy of daptomycin for the treatment of complicated skin and skin-structure infections. Clin Infect Dis 2004;38:1673–81. 15227611 10.1086/420818

[2] Fowler VG Jr Boucher HW Corey GR Daptomycin versus standard therapy for bacteremia and endocarditis caused by Staphylococcus aureus. N Engl J Med 2006;355:653–65. 16914701 10.1056/NEJMoa053783

[3] Levine DP Lamp KC Daptomycin in the treatment of patients with infective endocarditis: experience from a registry. Am J Med 2007;120:S28–33. 10.1016/j.amjmed.2007.07.011 17904948

[4] Baddour LM Wilson WR Bayer AS Infective endocarditis in adults: diagnosis, antimicrobial therapy, and management of complications a scientific statement for healthcare professionals from the American Heart Association. Circulation 2015;132:1435–86. 26373316 10.1161/CIR.0000000000000296

[5] Habib G Lancellotti P Antunes MJ 2015 ESC Guidelines for the management of infective endocarditis. Eur Heart J 2015;36:3075–128. 26320109 10.1093/eurheartj/ehv319

[6] Henter JI Horne A Aricó M HLH-2004: diagnostic and therapeutic guidelines for hemophagocytic lymphohistiocytosis. Pediatr Blood Cancer 2007;48:124–31. 16937360 10.1002/pbc.21039

[7] Li JS Sexton DJ Mick N Proposed modifications to the Duke criteria for the diagnosis of infective endocarditis. Clin Infect Dis 2000;30:633–8. 10770721 10.1086/313753

[8] Lee SY Fan HW Kuti JL Update on daptomycin: the first approved lipopeptide antibiotic. Expert Opin Pharmacother 2006;7:1381–97. 16805723 10.1517/14656566.7.10.1381

[9] Gould FK Denning DW Elliott TS Guidelines for the diagnosis and antibiotic treatment of endocarditis in adults: a report of the Working Party of the British Society for Antimicrobial Chemotherapy. J Antimicrob Chemother 2012;67:269–89. 22086858 10.1093/jac/dkr450

[10] Carugati M Bayer AS Miro JM High-dose daptomycin therapy for left-sided infective endocarditis: a prospective study from the international collaboration on endocarditis. Antimicrob Agents Chemother 2013;57:6213–22. 24080644 10.1128/AAC.01563-13 PMC3837915

[11] Guleri A Utili R Dohmen P Daptomycin for the Treatment of infective endocarditis: results from European Cubicin® Outcomes Registry and Experience (EU-CORE). Infect Dis Ther 2015;4:283–96. 26168988 10.1007/s40121-015-0075-9 PMC4575291

[12] Gould IM Miró JM Rybak MJ Daptomycin: the role of high-dose and combination therapy for Gram-positive infections. Int J Antimicrob Agents 2013;42:202–10. 23845504 10.1016/j.ijantimicag.2013.05.005

[13] Duval X Delahaye F Alla F Temporal trends in infective endocarditis in the context of prophylaxis guideline modifications: three successive population-based surveys. J Am Coll Cardiol 2012;59:1968–76. 22624837 10.1016/j.jacc.2012.02.029

[14] Correa de Sa DD Tleyjeh IM Anavekar NS Epidemiological trends of infective endocarditis: a population-based study in Olmsted County, Minnesota. Mayo Clin Proc 2010;85:422–6. 20435834 10.4065/mcp.2009.0585 PMC2861970

[15] Federspiel JJ Stearns SC Peppercorn AF Increasing US rates of endocarditis with Staphylococcus aureus: 1999–2008. Arch Intern Med 2012;172:363–5. 22371926 10.1001/archinternmed.2011.1027 PMC3314241

[16] Chu VH Woods CW Miro JM Emergence of coagulase-negative Staphylococci as a cause of native valve endocarditis. Clin Infect Dis 2008;46:232–42. 18171255 10.1086/524666

[17] Garcia-de-la-Maria C Marco F Armero Y Daptomycin is effective for treatment of experimental endocarditis due to methicillin-resistant and glycopeptide-intermediate Staphylococcus epidermidis. Antimicrob Agents Chemother 2010;54:2781–6. 20421394 10.1128/AAC.01011-09 PMC2897304

[18] Sass LA Ziemba KJ Heiser EA A 1-year-old with Mycobacterium tuberculosis endocarditis with mass spectrometry analysis of cardiac vegetation composition. J Pediatric Infect Dis Soc 2016;5:85–8. 26908495 10.1093/jpids/piu087

[19] Kiernan TJ O’Flaherty N Gilmore R Abiotrophia defectiva endocarditis and associated hemophagocytic syndrome: a first case report and review of the literature. Int J Infect Dis 2008;12:478–82. 18539495 10.1016/j.ijid.2008.01.014

[20] Wang Z Duarte AG Schnadig VJ Fatal reactive hemophagocytosis related to disseminated histoplasmosis with endocarditis: an unusual case diagnosed at autopsy. South Med J 2007;100:208–12. 17330695 10.1097/SMJ.0b013e31802b2812

[21] Naffaa M Awad J Oren I Group G streptococcal endocarditis-associated hemophagocytic syndrome. Int J Infect Dis 2013;17:e1237–9. 23816411 10.1016/j.ijid.2013.05.004

